# GeoBioScience: Red Wood Ants as Bioindicators for Active Tectonic Fault Systems in the West Eifel (Germany)

**DOI:** 10.3390/ani3020475

**Published:** 2013-05-17

**Authors:** Gabriele Berberich, Ulrich Schreiber

**Affiliations:** Department of Geology, Faculty of Biology, University of Duisburg-Essen, Universitätsstr. 5, 45141 Essen, Germany; E-Mail: ulrich.schreiber@uni-due.de

**Keywords:** red wood ants, *Formica rufa*-group, active tectonics, West Eifel (Germany), soil gas analyses, Helium, Radon, CO_2_, geostatistics

## Abstract

**Simple Summary:**

In a 1.140 km² study area of the volcanic West Eifel, approx. 3,000 Red Wood Ant (RWA; *Formica rufa*-group) mounds had been identified and correlated with tectonically active gas-permeable faults, mostly strike-slip faults. Linear alignment of RWA mounds and soil gas anomalies distinctly indicate the course of these faults, while clusters of mounds indicate crosscut zones of fault systems, which can be correlated with voids caused by crustal block rotation. This demonstrates that RWA are bioindicators for identifying active fault systems and useful where information on the active regime is incomplete or the resolution by technical means is insufficient.

**Abstract:**

In a 1.140 km² study area of the volcanic West Eifel, a comprehensive investigation established the correlation between red wood ant mound (RWA; *Formica rufa*-group) sites and active tectonic faults. The current stress field with a NW-SE-trending main stress direction opens pathways for geogenic gases and potential magmas following the same orientation. At the same time, Variscan and Mesozoic fault zones are reactivated. The results showed linear alignments and clusters of approx. 3,000 RWA mounds. While linear mound distribution correlate with strike-slip fault systems documented by quartz and ore veins and fault planes with slickensides, the clusters represent crosscut zones of dominant fault systems. Latter can be correlated with voids caused by crustal block rotation. Gas analyses from soil air, mineral springs and mofettes (CO_2_, Helium, Radon and H_2_S) reveal limiting concentrations for the spatial distribution of mounds and colonization. Striking is further the almost complete absence of RWA mounds in the core area of the Quaternary volcanic field. A possible cause can be found in occasionally occurring H_2_S in the fault systems, which is toxic at miniscule concentrations to the ants. Viewed overall, there is a strong relationship between RWA mounds and active tectonics in the West Eifel.

## 1. Introduction

**Figure 1:**
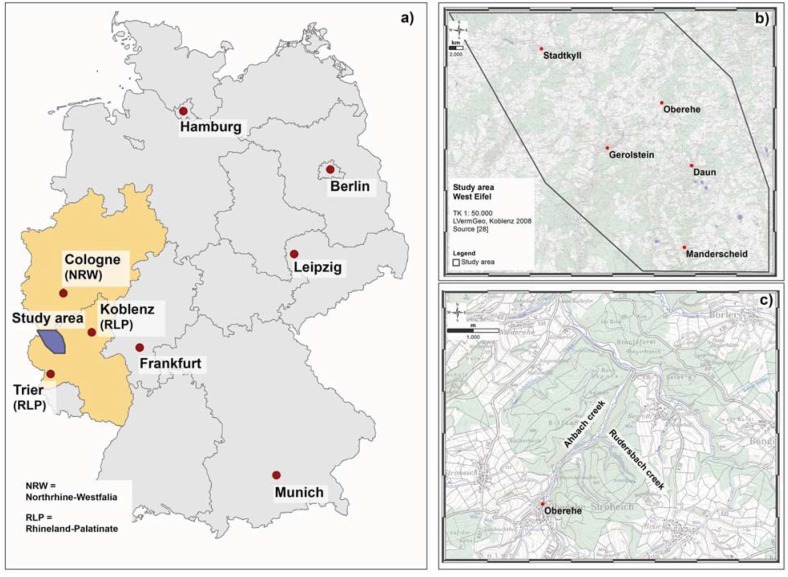
Position of the 1,140 km² study area within Germany (**a**), locations of the study area within the West Eifel (**b**) and the reference location Oberehe (**c**).

The 1,140 km² study area (*cf.* Figure 1(a,b)) with its reference location Oberehe (*cf.* Figure 1(c)) is located in the volcanic West Eifel (approx. 100 km SW of Cologne, Germany). Though under geological and structural investigation for more than 200 years, only spatially limited information on active fault systems are available at present. In a detailed and statistically well based study, it was investigated whether a GeoBioScience-approach can be used to correlate and identify tectonically active fault systems in the 1,140 km² study area in the West Eifel. This approach included the mapping of RWA mounds and their spatial distribution, identification of gas anomalies, mapping of tectonic features and statistical analyses.

The study area is composed of a several thousand meter thick sequence of Lower Devonian clastic sediment rocks. The “Eifel North-South Zone”, a north south facing depression zone, preserved Middle Devonian sediment rocks in predominantly carbonate facies. Triassic sediment rocks are found in the area between Gerolstein and Hillesheim [1]. Commenced during the Paleogene more than 100 volcanoes erupted at the eastern flank of the “Eifel North-South Zone”. A second volcanic phase producing more than 270 eruption centers started in the Quaternary. The most recent eruption dates back 11,000 years [1,2]. The study area has a complex tectonic history. At the beginning of the Carboniferous marine sediments underwent the Variscan Orogeny, resulting in the formation of the basic structure of the Rhenish Massif characterized by NE-SW trending folds and thrusts [3]. Changes in the intraplate compressional stresses exerted on continental Europe lead to the formation of a shear system during the Mesozoic [4]. During the Cenozoic, presumably from Eocene to Miocene, the main stress regime rotated from NNE-SSW to NW-SE direction in the Rhenish Massif. This caused a NE-SW extension in combination with a clockwise block rotation, a re-activation and re-organization of postvariscan strike-slip faults in approximately WNW-ESE direction and the formation of the tectonic depressions, *i.e.*, the Neuwieder Basin and small-scale transtension zones [5]. The WNW-ESE trending strike-slip faults are staggered in equidistant intervals of several kilometers. This system continues from the Eifel to the North into the Ruhr Carboniferous, where it has been recognized due to the extensive underground coal mining first [6]. This situation has led to a complex tectonic break clod. The progressive uplift and the SW-drift of the Western part of the Rhenish Massif, commenced in the early Miocene are still persisting during the Quaternary. The uplift is mainly attributed to plume-related thermal expansion of the mantle-lithosphere and tectonic stress [7,8,9,10,11,12]. Ongoing geotectonic processes still influence the active regional tectonics and lead to a moderate seismicity [13,14]. Currently, the present NW-SE main stress direction opens pathways for migration of geogenic gases and potential magmas [7,15,16,17]. In the West Eifel and Hocheifel, very CO_2_ gas-rich mineral waters show significantly high concentrations of lower crustal-derived or upper mantle-derived chemical tracers, such as Helium (He), Radon (Rn) and Carbon dioxide (CO_2_). They are commonly interpreted as a result of magmatic or tectonic processes in the Earth’s upper mantle and lower crust caused by volcanism, crustal updoming, and rift processes [18,19,20,21].

At present, only limited information on active fault systems are available: eruption fissures of the Quaternary volcanic field provide information on the course of active fault systems striking in W-E (80°; degrees from N), NW-SE (120°–140°) and N-S (170°–180°) directions [22,23]. Lineament analysis between Gerolstein and Daun show two main tectonic directions: NNW-SSE (±170°) and WNW-ESE (100°–110°) [24]. Statistical analyses [25] applied on the spatial distribution of the directions of fault zones [26,27], 210 mineral springs and mofettes [28] and earthquake epicenters [13,14] showed that the centers of the modes, *i.e.*, the local maxima in the histograms, denote the preferential alignment directions, while their widths indicate the corresponding directional variations. The spatial distribution of the tectonic features follows the opening direction of the Quaternary volcanic field and shows active ±NW-SE, NE-SW and ±WNW-ESE striking fault directions [29,30,31]. Barite dykes set-up in the Variscan period and reactivated in the Cenozoic at the NE edge of the study area indicate a NNE-SSW sinistral strike-slip fault system [32]. Lead, copper, silver and quartz bearing dykes at the NE, SE and NW edge of the study area indicate NW-SE, NE-SW and N-S fault directions as well [33,34,35].

**Figure 2:**
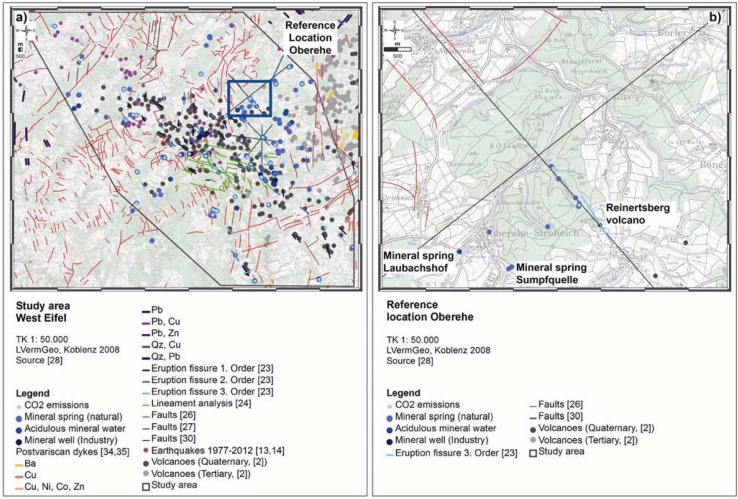
Tectonic features of the 1,140 km² study area (**a**) and of the reference location Oberehe (**b**) according to the current state of knowledge. At Oberehe it is visible, that knowledge on the tectonic regime [26,30] is limited and incomplete due to the coverage of tectonic faults by soil cover and vegetation (forest stands).

The reference location Oberehe (*cf.* Figure 2(b)) is located SE of the Hillesheimer Depression (Devonian Klerf strata). A volcanic eruption fissure along the Rudersbach creek strikes approximately NW-SE (±140°), measured slickensides and a linear arrangement of mineral springs (Rudersbach creek) give indications of active fault structures [23,28,29]. Other mineral springs rise at the Oberehe parking lot SW of Oberehe (Laubachshof), around the so-called Sumpfquelle (SE of Oberehe) and along the Rudersbach creek. Nevertheless, knowledge on the active tectonic regime is limited and incomplete due to the coverage of tectonic faults by soil and vegetation (forest stands). 

**Figure 3:**
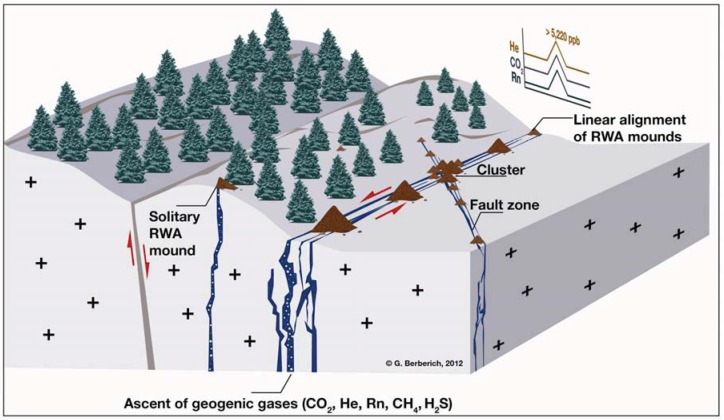
GeoBioScience: Relationship between RWA mound sites and gaspermeable tectonic fault systems showing gas anomalies: Along the open systems geogenic gas concentrations exceed atmospheric standards (He > 5,220 ppb) and background values (CO_2_ > 2 Vol%; Rn, CH_4_ and H_2_S depending on the geological setting). Linear alignment of RWA mounds occurs if the gaspermeable fault is running open for a very long distance, e.g., 3 km at the Oberehe site (West Eifel, *cf.* Figure 5(b)). At crosscut zones of tectonic fault systems clusters of RWA mounds occur. Solitary mounds can be found mostly at unusual locations with spotty degassing [28].

Red wood ants (RWA; *Formica rufa*-group (Hymenoptera: *Formicidae*)) are useful bioindicators for the identification of active tectonic fault systems (*cf.* Figure 3) [28,36]. A particular advantage of the RWA is their high sensitivity to environmental changes. Forced by some million years of evolutionary selection they developed anticipatory mechanisms. Besides an extremely strong temperature sensitivity (0.25 K), they have chemo-receptors for the detection of CO_2_-concentrations and an electromagnetic field sensitivity [37,38,39,40,41,42]. As early as the beginning of the last century, entomologists presumed a dependency of spatial RWA mound distributions on geological formations [43,44,45]. Work [46] already stated in 1929 that there are no preferred locations of RWA mounds within a forest stand. Typical in-line alignments of RWA mounds regardless of any preferred direction, *i.e.*, edge effects of forest stands and/or roads, were described for the nature reserve Rudolfshagen [47]. Consequently, an integrated GeoBioScience-approach requires the consideration of the interaction between the abiotic (e.g., tectonic processes) and biotic (e.g., preferred settlement sites by RWA) environment.

## 2. Materials and Methods

**Figure 4:**
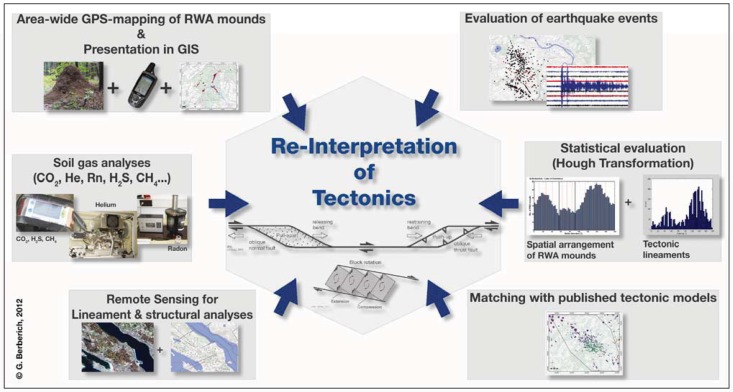
In a comprehensive GeoBioScience-approach, different methods were combined and applied to investigate the relationship between RWA mounds and active tectonic fault systems.

In the study area and at the reference location Oberehe, a comprehensive investigation was successfully conducted in order to investigate the correlation between RWA mounds and an active tectonic fault regime. Therefore a combination of gas analyses (soil gas and gas of mineral springs and mofettes), area-wide GPS-mapping of RWA mounds to identify distribution pattern, evaluation of earthquake events and structural analyses were carried out (*cf.* Figure 4). This GeoBioScience-approach can be used to further understand settlement processes of RWA and to complement the knowledge of the active tectonic fault regime in the volcanic West Eifel.

### 2.1. Mapping and Data Collection

The area-wide investigation was carried out in the 1.140 km² study area of the volcanic West Eifel (*cf.* Figure 2(a)). From August 2008–September 2009, all inhabited RWA mounds were mapped using a Garmin 60CSx GPS receiver. Additionally the ecological habitat requirements, such as altitude, exposition, inclination, vegetation (type of forest stands), site moisture, and their current height and diameter were determined (using the active exit openings as borderlines of the inhabited mounds) [43,46,48,49,50]. All collected information was then incorporated to a mound site database. In addition, the ant species was determined [51].

### 2.2. Gas Samples and Analyses

The soil gas analyses were focused on the concentration of Carbon dioxide (CO_2_), Helium (He), Radon (Rn) and Hydrogen sulfide (H_2_S). CO_2_ serves as carriers for other gases (e.g., He and Rn). Helium is confirmed as an outstanding fault indicator. Crustal degassing along faults is indicated by He anomalies, whereas Rn as a tracer provides a qualitative measure of gas migration. H_2_S as a minor or trace component is supposed to indicate hydrothermal influence or degassing processes at volcanoes [52,53,54,55,56,57]. 

Soil gas samples were collected by pounding a stainless steel probe (with a sacrificial tip) to the desired depth of 1.0 m into the unsaturated zone. It is assumed that meteorological influences can be excluded at this depth [58]. All samples were analyzed for their content of He, Rn, CO_2_ and H_2_S. After the probe was driven into the ground, it was fitted with an airtight cap and septum for withdrawal of the soil-gas sample. Before removal of the first sample, 40 mL air was withdrawn from the probe to remove air introduced when the probe was emplaced in the ground. Samples were then collected from the hollow probe by inserting the needle of a syringe through the septum in the cap and withdrawing 20 mL of the soil gas. It was analyzed for He immediately in the field to exclude He migration out of the sample and intrusion of atmospheric air. A mobile, modified mass spectrometer (Alcatel ASM 142; adixen) that had been converted to a 20 mL sample volume for a single He-measurement was run on-site. Standard samples of air were analyzed to ensure stability of the instrument. As the sample can be diluted by atmospheric air when being transferred from the syringe into the mass spectrometer, the measured residual He concentration was corrected accordingly [59]. For Rn, a 100 mL sample was transferred in an evacuated, 100 mL volume ZnS(Ag)-plated Lucas cell by inserting the needle of the syringe containing the gas sample through the septum of the evacuated cell and allowing the sample in the syringe to be drawn inside. To allow the radiation to take place, the sample was analyzed after three hours minimum using a Lucas detector (JP048; Radon Detector LUK4). To obtain a semi-quantitative measure of the gas leakage in the study area (CO_2_, CH_4_ and H_2_S), a portable Dräger-meter equipped with three sensors with different detection limits was used over a measuring time of two minutes (Dräger X-am^®^ 7000; DrägerSensor^®^ Smart IR CO_2_ HC, measuring range 0–100% by volume, DrägerSensor^®^ Smart XS EC H_2_S 100 ppm; range 0–100 ppm H_2_S and DrägerSensor^®^ IR Ex HC CH_4_; lower detection limit 225 ppm). Collected and measured data were computer processed using Excel 2010 for statistical calculations and diagrams. Spatial data analysis (ArcGIS 10) including the Analysis Tools Extensions was used to study the variables and their relationship. Gas samples from mineral springs and mofettes were performed using a “gas-mouse”, which consists of a septum and a 100 mL PVC bottle with an airtight connected funnel. The gas-mouse and its funnel were total flooded with water. Degassing CO_2_ bubbles were trapped by the funnel and collected in the PVC bottle displacing the water. To get a representative gas sample degassing bubbles at different locations were captured. Then the He and Rn samples were collected and the concentration of CO_2_, H_2_S and CH_4_ were determined following the soil gas sampling procedure. 

## 3. Results and Discussion

### 3.1. Spatial Distribution Pattern of RWA in the Study Area and at the Reference Location Oberehe

**Figure 5:**
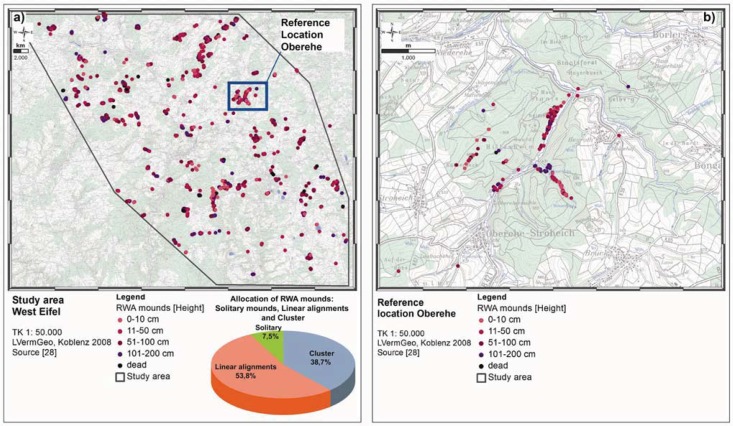
Spatial RWA mound distribution (approx. 3,000 mounds) in the 1,140 km² study area (**a**) and at the reference location Oberehe (**b**). In the study area, RWA mounds are not statistically distributed, but follow a certain shaped pattern: Roughly, 54% are in linear arrangements with a length ranging from several meters to kilometers, 39% of the mounds are found in clusters and approx. 7% are solitary mounds that appear in different habitats. At the reference location Oberehe (b) 212 RWA mounds predominantly in linear alignment were mapped. Though the reference site is surrounded by various forest stands, no further RWA mounds were found in those forest stands during the area-wide mapping.

Around 43% of the study area is forested (490 km²) and 57% (650 km²) are fields, meadows, roads, paths and built-up areas. In total, approx. 3,000 RWA mounds have been mapped mainly (74%) in forest stands (*cf.* Figure 5(a)). Approximately 7% are solitary mounds that appear in different habitats. Roughly, 54% are in linear arrangements with a length ranging from several meters to kilometers. 39% of the mounds are found in clusters of an average of 50 mounds on a limited area of a few hectares. Overall, 22 clusters could be identified in the study area: nine of them cover an average area of 1.0 ha, seven of them 6.7 ha and six of them larger areas (>15 ha). Particularly noticeable is the fact that clusters appear exclusively in forest areas with an average distance of about 5 km between them. Predominantly agricultural areas are not suitable sites. The clusters are structured and can be divided into linear arrangements (72%), clusters (15%), and solitary mounds (13%).

At the Oberehe site (*cf.* Figure 5(b)), 212 RWA mound predominantly in linear alignment were mapped: 116 RWA mounds along the 3 km Ahbach creek, at the Kälberheck ridge 31 RWA mounds roughly parallel in several linear alignments and 65 RWA mounds along the Rudersbach creek. Though the reference site is surrounded by various forest stands, no further RWA mounds were found in those forest stands during the area-wide mapping.

**Figure 6:**
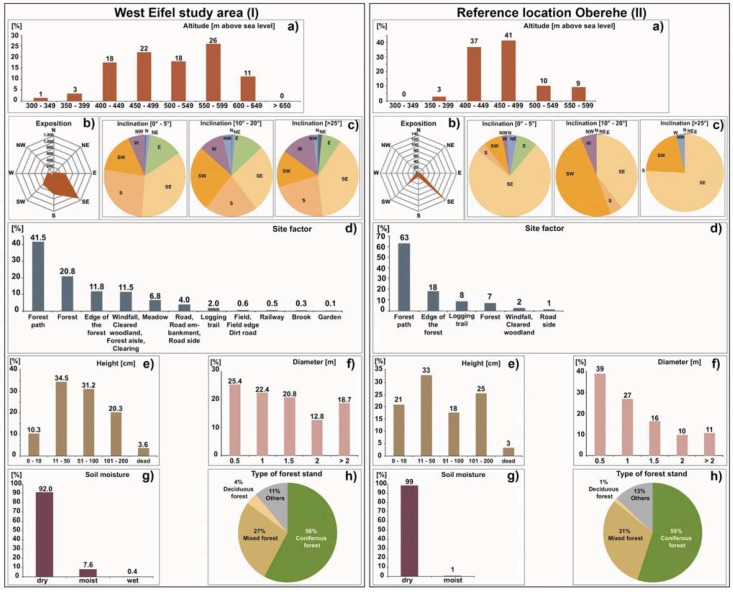
Comparison of the habitat requirements of the RWA mounds for the entire study area (**I**) and reference location Oberehe (**II**). Given numbers are for altitude (m above sea level) (**a**), exposition [°] (**b**), inclination [°] (**c**), site factor (**d**), height [m] (**e**), diameter [m] (**f**), site moisture (**g**) and type of forest stand (**h**).

A comparison of the habitat requirements of the RWA mounds for the entire study area and the reference location Oberehe confirmed the published ones so far [43,44,45,46,48,49,50]. Between 80% and 84% of the mapped mounds were found at altitudes of between 400 and 600 m above sea-level (*cf.* Figure 6(a)). Most of the RWA mounds have a very strong SE orientation, though there is also a weak SW orientation visible (*cf.* Figure 6(b)). In the study area and at the Oberehe site, the preferred slope angles are very shallow (0–5°) (*cf.* Figure 6(c)). Only 7% (study area) and 10% (Oberehe) of the total mounds were mapped at very steep slopes (>25°) with SE orientation. In the study area and at the Oberehe site the majority of mounds (99%) were mapped in forest stands (at forest roads, forest edges, logging trails, cleared woodland (*cf.* Figure 6(d)). Only 1% of the RWA mounds occur in unusual, non-typical locations such as road sides, embankments, creeks or private gardens. Although these sites do not meet the site requirements published in the literature so far, the mapped mounds of these unusual locations are usually several years old as confirmed by forest workers or house owners. Around one third of the mounds (34% study area, 33% Oberehe) show mound heights between 0.11 and 0.50 m. Only a fifth (20% study area) shows heights between 1.0 and 2.0 m, whereas at the Oberehe site 25% have heights between 1.0 and 2.0 m (*cf.* Figure 6(e)). Approximately 43% of the mounds have diameters between 1.0 and 1.5 m. However, 31.5% of the mound in the study area and 21% (Oberehe) have exceptionally large diameters of 2 m and larger (*cf.* Figure 6(f)). The majority of the mounds were found at dry sites (92% study area, 99% Oberehe); only a minority (8% study area, 1% Oberehe) was found on humid or wet locations (*cf.* Figure 6(g)). The majority of the mounds was mapped in coniferous forest stands, e.g., picea (58% study area, 55% Oberehe), approx. one third was found in mixed forest stands (e.g., picea, fagus and/or quercus) and very few were found in deciduous (fagus and/or quercus) forests (4% study area, 1% Oberehe) (*cf.* Figure 6(h)).

To capture the long-term population dynamics in the entire study area, recent mapping of RWA was compared with previous RWA mappings [60,61,62]. As the quality of mapping varied in the past, the total number of mounds in the mapped area can be considered a first approximation (*cf.* Table 1). A total of 562 RWA mounds were mapped in the Daun region between 1960 and 1968 [60]. Today, there are 988 RWA mounds in the same area, *i.e.*, almost two times more than 50 years ago. A total of 356 RWA mounds were mapped 1984 in the Kelberg and Adenau region [61]. Now there are 1,408 mounds in the same area, nearly four times more than 30 years ago. In 1991, a rough mapping of Forest Office Salm identified 66 RWA mounds in the Salm forest [62]. Today there are almost twice as many (128) mounds. Overall, it can be said that the number of RWA mounds in the study area has increased significantly in the past few decades. This finding is consistent with own findings at the Lake of Constance and observations described in other German regions, e.g., the Free State of Saxony and contradictory to the common tenet that RWA should be classified as endangered species as defined by the BfN-Red List of Threatened Species [63,64,65,66,67]. 

**Table 1:** Analyses of the long-term population dynamics in the entire study area. Recent mapping of RWA mounds was compared with previous RWA mappings. The number of RWA mounds in the study area has significantly increased in the past few decades. This finding is consistent with observations described in other German regions and contradictory to the common tenet that RWA should be classified as endangered species as defined by BfN-Red List of Threatened Species [63,64,65,66,67].

Author	Year	Area	Sum	Recent mapping [28]	Factor
Wellenstein [60]	1960–1968	Daun region	562	988	1.75
Moelter [61]	1984	Kelberg, Adenau	356	1,408	3.95
Forest Office Salm [62]	1991	Salm Forest	66	128	1.94

**Figure 7:**
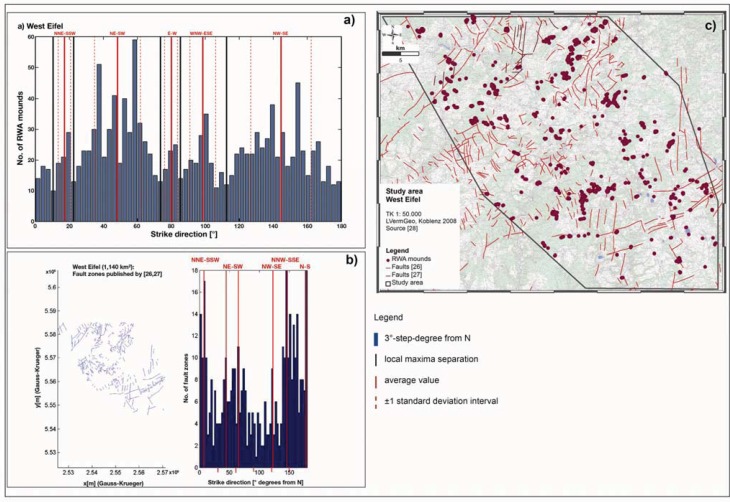
Hough transform analyses of spatial distribution of RWA mounds (degrees from N) in the 1,140 km² West Eifel study area (**a**) in comparison with fault zones (degrees from N) (**b**) and in a map presentation (**c**) show that the RWA mounds are not randomly distributed but that there are preferential alignment directions of RWA mounds that correspond to those of the main fault directions.

Furthermore, the spatial distribution of RWA mounds shows that they are not statistically randomly distributed, but follow a certain shaped pattern [28,31]. This could be verified by the Hough transform (a well-established algorithm for identifying linear structures in sets of points [25]) applied on the spatial distribution of RWA mounds in entire the study area (*cf.* Figure 7(a)) in comparison with fault zones [26,27] (*cf.* Figure 7(b)). Statistical analyses show that the center’s of the modes, *i.e.*, the local maxima in the histograms, denote the preferential alignment directions, while their widths indicate the corresponding directional variations. In both histograms (*cf.* Figure 7(a,b)), the local maxima are separated by vertical black lines. For each mode, the average value is indicated by a solid vertical red line combined with direction (*i.e.*, NW-SE) and the ±1 standard deviation interval surrounded by two dashed vertical red lines. Each blue bar represents a 3°-step-degree from N. More specifically, the azimuthal directions in which the RWA mound positions are aligned correspond to those of the main fault directions. 

In the West Eifel, the large-scale arrangement of RWA mounds (*cf.* Figure 7(a)) depicts mainly the opening direction of the Quaternary volcanic field (NW-SE; 120°–140° degrees from N) and the associated WNW-ESE (100°–110°) regime as well as the reactivated Variscan fault systems (NNE-SSW; approx. 20° which indicate block rotation), NE-SW (approx. 50–60°) and the N-S (170°–180°) direction of the “Eifel North-South Zone” [31].

### 3.2. Geogenic Gas Concentrations in the Study Area and Anomalies

A total of 58 gas analyses were performed in mineral springs and mofettes in the West Eifel study area. Additionally, at the Oberehe site, a total of 128 soil gas analyses served to investigate whether there is a correlation of RWA mounds and gas anomalies.

#### 3.2.1. Gas Concentrations in Minerals Springs and Mofettes of the Study Area

**Figure 8:**
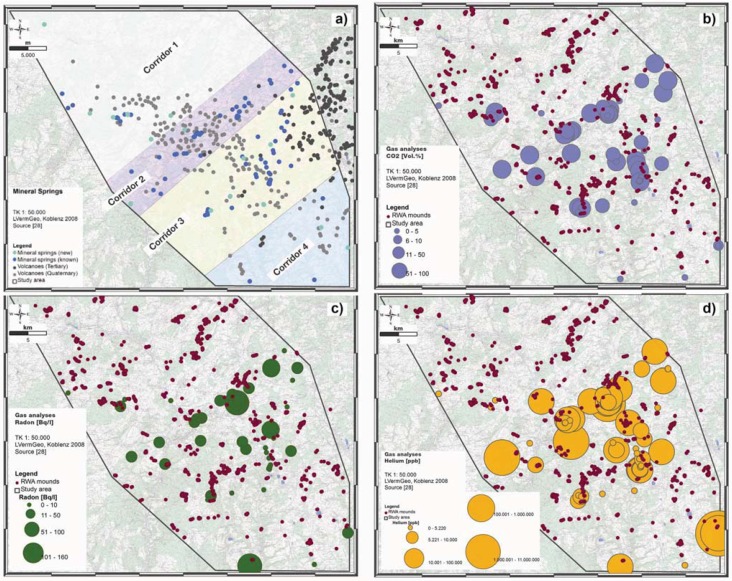
West Eifel study area: Spatial distribution pattern of known (dark blue dots) and new minerals (light blue dots) springs within the Quaternary volcanic field (**a**) as well as CO_2_ (blue dots) (**b**), Radon (green dots) (**c**) and Helium (orange dots) concentrations (**d**) in mineral springs and spatial distribution of RWA mounds (dark red dots; (b), (c), (d) [28]). Increasing gas concentrations are shown by increasing dots.

In the West Eifel study area, 210 mineral springs were mapped. A total of 125 (59.5%) mineral springs were remapped: 26 new ones which show CO_2_ emissions, 95 are natural mineral springs and four are natural mineral springs with CO_2_ emissions. The mineral springs occur from North to South in four NE-SW trending spring corridors (*cf.* Figure 8(a)), from which the both corridors in the center of the Quaternary volcanic field are the most obvious [28]. Results of the Hough transform applied to the spatial distribution of mineral springs and mofettes correspond to those of the main stress regime: the opening direction of the Quaternary volcanic field (NW-SE), and the associated WSW-ENE extensional regime and the reactivated Variscan fault systems (NNE-SSW, NE-SW) [31].

Gas analyses of minerals springs performed in December 2008 and January/April 2009 showed a mean CO_2_ concentration of 59 Vol% and maximum concentration of 84 Vol%. The atmospheric standard is 0.0370 Vol%. The data distribution is unbalanced throughout the study area, as suggested by the dissimilarity of the mean (59 Vol%) and the median values (73 Vol%). A correspondence to atmospheric content is given by the lower quartile value (0.043 Vol%). The upper quartile value (80 Vol%) corresponds to the anomaly threshold. Elevated outlier CO_2_ values shift the data distribution toward higher concentrations, as suggested by the low skewness. The highest CO_2_ concentrations (>70 Vol%) were determined in around 40% of sampled mineral springs. It turns out that the highest CO_2_ concentrations occur in the center part of the study area; in both NE-SW trending spring corridors 2 and 3 ((*cf.* Figure 8(b)). 

The Rn concentrations in minerals springs showed a mean concentration of 25 Bq/L and median value of 10 Bq/L with a maximum concentration of 152 Bq/L in the Dreiser Weiher site ((*cf.* Figure 8(c)). The radon concentrations reflect, similar to the CO_2_ concentrations the spring corridors 2 and 3. Corridor 2 showed somewhat higher radon concentrations compared to corridor 3, but both corridors are clearly depicted by the radon levels. Spring corridor 1 and 4 are only diffusely indicated.

Helium concentrations in minerals springs are generally above average, as given by the mean (256,884 ppb He) and median value (8,357 ppb He). Very high Helium values were measured in spring corridor 2 (Geeser Dress with approximately 170,000 ppb He, in the Dreiser Weiher (approximately 161,000 ppb He) and in the Büdesheimer Drees (approximately 104,000 ppb He). The highest He concentration at all was detected at the Strotzbüscher Mühle at the SE edge of the study areas (*cf.* Figure 8(d)). Here peak concentrations around 10,500,000 ppb (equivalent to about 1.0% He) were determined. Thus, the underlying background level of 5,220 ppb is exceeded many times over. 

The wide ranges, as well as the high values of the skewness, for He, Rn and H_2_S concentrations indicate the presence of outliers. The mean and the median values for He, Rn and H_2_S highlight a positively skewed frequency distribution of these gases and indicate an exponential or lognormal distribution of these variables.

The gas concentrations of the minerals spring clearly depict two stress directions: the NW-SE trending degassing lines characterize the NW-SE-trending opening direction of the Quaternary volcanic field but also the reactivated Variscan fault systems in NE-SW direction. There is a good correspondence between geochemical data results and the ones of the statistical analyses [31].

#### 3.2.2. Gas Concentrations and Anomalies at Oberehe

**Figure 9:**
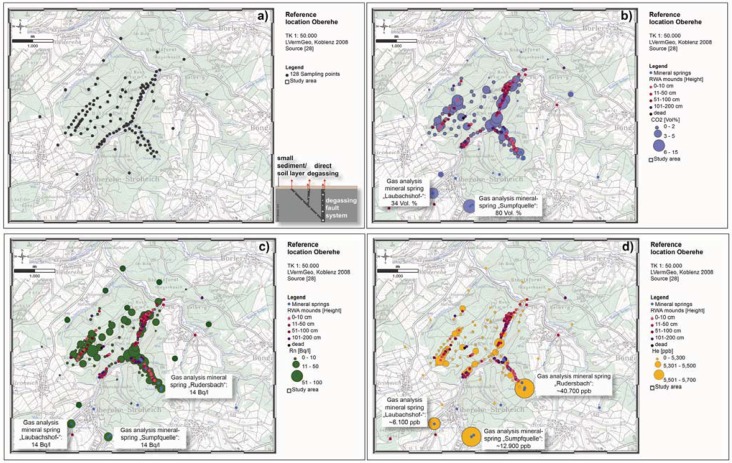
Relationship between soil gas anomalies and alignment of the RWA mounds for the Oberehe site (**a**) showing sampling points (black dots), (**b**) CO_2 _anomalies (>2 Vol%; blue dots and mineral springs (small blue dots)), (**c**) Radon anomalies (up to 74 Bq/L; green dots) and (**d**) Helium anomalies (He > 5,220 ppb; orange dots). In addition, the concentrations of the three gases in mineral springs are shown.

In 2009 and 2012, 128 soil gas analyses (*cf.* Figure 9(a)) were carried out at the reference location Oberehe (Klerf schists of the Middle Devonian) in order to evaluate the relationship between active tectonics, geogenic degassing structures and spatial RWA distribution pattern. Therefore, it was the objective to measure the geogenic gas concentrations in close proximity to the mounds to get as much information on the degassing structures as possible. In areas without RWA mounds control samplings were performed (in 1 km distance). Compared to their atmospheric concentrations (0.0370 Vol% CO_2_, 5,220 ppb He, 0.01 kBq/m^3^ Rn, 1.5 ppm CH_4_ and 0.002 ppm H_2_S) and the regional average background values for Helium (5,202 ppb [28]) and Rn (46 Bq/L [56]), the soil gases showed different concentrations (*cf.* Table 2). Neither CH_4_ nor H_2_S were present. 

Statistical analyses of the soil gas data showed a mean CO_2_ concentration of 2 Vol% and a maximum concentration of 14 Vol%. The data distribution is rather symmetric throughout the study area, as suggested by the similarity of the mean (2 Vol%) and the median values (1 Vol%). The highest CO_2_ concentrations were found in the floodplain of the Ahbach (about 14 Vol%) and on the ridge of the Kälberheck (around 7 Vol%). CO_2_ concentrations in soil gas indicate two NNE-SSW (±15°) striking fault zones along the floodplain of the Ahbach and on the ridge of the Kälberheck. Furthermore, a NW-SE (±140°) striking fault zone along the Rudersbach is indicated by CO_2_ concentrations up to 5 Vol% in soil gas (*cf.* Figure 9(b)). This active fault zone was already identified by a volcanic eruption fissure and a linear arrangement mineral springs [23,29,30].

**Table 2:** Main statistical parameters of gas concentrations in the study area (mofettes and mineral springs) and at the reference location Oberehe (soil gas) (N = number of samples; LQ = lower quartile range; UQ = upper quartile range; IQR = interquartile range, StD = Standard deviation).

	Entire study are (gas analyses from mofettes and mineral springs) [28]					Reference location Oberehe (soil gas analyses) [28]				
	CO_2_ [Vol%]	He [ppb]	Rn [Bq/L]	CH_4_ [ppm]	H_2_S [ppm]	CO_2_ [Vol%]	He [ppb]	Rn [Bq/L]	CH_4_ [ppm]	H_2_S [ppm]
N	42	58	55	58	3	126	127	128	128	128
Mean	59	256,884	25	0	4	2	5,247	13	0	0
Median	73	8,357	10	0	2	1	5,236	9	0	0
Min.	2	2,033	0	0	2	0	4,941	0	0	0
Max.	84	10,523.427	152	0	8	14	5,636	74	0	0
LQ	43	3,835	5	–	2	0	5,197	3	–	–
UQ	80	21,190	33	–	5	2	5,276	18	–	–
IQR	37	17,355	28	–	3	1	80	60	–	–
StD	25	1,418.216	34	–	3	2	97	15	–	–
Skewness	-1	7	2	–	2	3	1	2	–	–
Atmospheric standard	0.037	5,220	0.01	1.5	0.002	0.037	5,220	0.01	1.5	0.002
Average regional background value	–	–	–	–	–		5,202 [28]	46 [56]	–	–

The regional mean Rn concentration in soil gas is 46 Bq/L, the maximum 143 Bq/L [68]. The highest radon concentrations are found in the floodplain of the Ahbach (74 Bq/L and 69 Bq/L), in the area where the Rudersbach runs into the Ahbach (72 Bq/L) and at one site on the Kälberheck ridge (69 Bq/L). Five sampling sites showed concentrations above 50 Bq/L. Anomalous CO_2_ values (between 3 and 10 Vol%) correlate well with high Rn anomalies (up to 74 Bq/L) suggesting a fit with supposed local fault systems and a radon transport by ascending CO_2_. This indicates tectonic shearing of the rocks during the tectonic movement, with an associated increase in permeability [56]. The NE-SW (±50°), WNW-ESE (±110°), NW-SE (±140°) and the two NNE-SSW (±15°) striking faults are also well indicated by the radon concentrations. The radon concentrations of the degassing mineral springs are low at about 14 Bq/L (*cf.* Figure 9(c)). 

The soil gas data of the outstanding fault zone tracer He showed a mean concentration of 5,247 ppb exceeding the atmospheric standard of 5,220 ppb [69]. The data distribution is rather symmetric throughout the study area, as suggested by the similarity of the mean (5,247 ppb He) and the median values (5,236 ppb He). A correspondence to atmospheric content is given by the lower quartile value (5,197 ppb). The upper quartile value (5,276 ppb) corresponds to the anomaly threshold [55]. About 60.5% of the samples have significantly increased He concentrations (between 5,221 ppb and 5,635 ppb He), providing evidence of degassing anomalies (*cf.* Figure 9(d)). The helium concentrations in the mineral springs of the Rudersbach, the Sumpfquelle and Laubachshofquelle are between ≈6,100 and ≈40,700 ppb. They significantly exceed the atmospheric helium standard of 5,220 ppb roughly 8-fold [69]. At Oberehe the degassing structures are not diffuse but rather channeled along deep reaching pathways. They are comparable to those recorded for the Latera geothermal field or intramontane basins (Italy) [53,54]. The helium anomalies clearly reflect the fault zones NNE-SSW and NW-SE striking that have already been identified by their CO_2_ concentrations. In addition, a NE-SW (±50°) striking fault zone is also indicated. At certain locations in the Ahbach floodplain, elevated CO_2_concentrations appear (≈5–7 Vol%) along with helium anomalies (≈5,340–5,380 ppb). Such a relationship is interpreted as an indication of deep reaching fault zones [70].

It is striking that all soil gas concentrations of CO_2_, Rn and the fault zone tracer He are below atmospheric standards in the surrounding forest stands where also no RWA mounds were mapped. This indicates particularly favorable settlement conditions for RWA. Their mounds can only be found where geogenic gas anomalies occur. The comparison of both the spatial RWA mound distribution with the significantly increased gas anomalies confirms that the RWA colony is situated in a tectonically sheared region.

#### 3.2.3. Correlation of RWA Mounds and Gas Concentrations at the Reference Site Oberehe

Approximately 60% of the mounds are located in areas with Helium concentrations above-the atmospheric standard (5,220 ppb) and approximately 33% of the mounds are located in areas where radon anomalies (20–74 Bq/L) are found. 22% of the mounds are located at CO_2_ concentrations of 5–14 Vol%. CO_2_ concentrations above 8–10% that have a sufficiently high gas flux rate to cause an impact on the ecosystem [71]. Nevertheless, RWA mounds, especially mound start-ups (height 0–10 cm) can only be found at these locations with an enhanced CO_2_-degassing (*cf.* Figure 9(b)). At high CO_2_ gas flux rates, there might be no need for larger mounds dimensions (heights more than 10 cm) to trap CO_2_ and to positively influence and support the respiration metabolism of pupae [72] or the discontinuous breathing [38,73]. Furthermore, the RWA’s ability to detect CO_2_-gradients may provide an evolutionary advantage in site-selection and mound start-up processes [39]. In conclusion, soil gas anomalies indicate particularly favorable settlement conditions for RWA. The specific mechanisms how RWA find and why RWA prefer such sites with an enhanced degassing need to be researched in in-situ experiments and in close cooperation with myrmecologists in future within a well defined research project.

**Figure 10:**
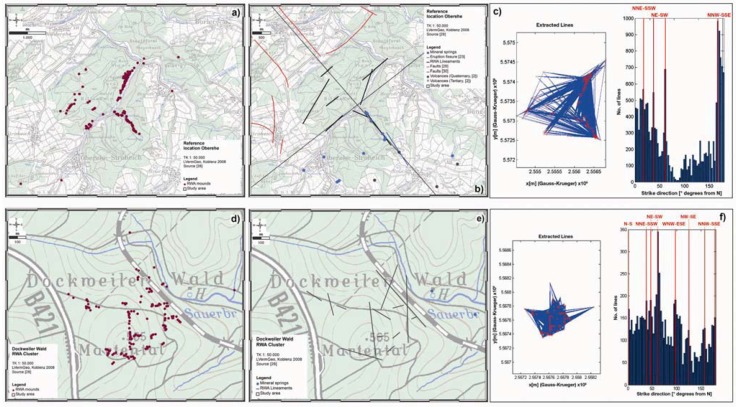
Spatial distributions of RWA (red dots) (**a**), manually inferred RWA Lineaments (grey lines) (**b**) combined with published tectonic features like faults (red lines), eruption fissures (blue lines) and mineral springs (blue dots) and results of the modified Hough Transform (**c**) for the Oberehe site. For the RWA cluster at the Dockweiler Wald area (**d**), manually inferred RWA lineaments (grey lines) and mineral springs (blue dots) (**e**) and results of the modified Hough Transform (**f**) are presented.

At the Oberehe site (*cf.* Figure 10(a)), the most preferential alignment directions of RWA Lineaments (*cf.* Figure 10(b)) manually inferred from the tectonic history, geological field work and experiences, and soil gas sampling are the reactivated Variscan fault systems NNE-SSW (approx. 20°) and NE-SW (40°–50°) and the opening direction of the Quaternary volcanic field in NW-SE (approx. 145°) direction. This result correlates well with a modified Hough transform method [74] applied on the RWA mound distribution (*cf.* Figure 10(c)). In two consecutive steps, all possible directions were extracted from the RWA mound positions first. For each mound pair a corresponding line was constructed. Then the distance of all mounds from the line was computed. Hereafter, the orientation of the extracted directions were binned in a histogram. The most preferential alignment directions are NNE-SSW (18°), NW-SE (171°) and strikingly NE-SW (39°–63°). The NE-SW direction might be caused by small-scale rotation of the stress field due to block rotation. Clusters of RWA mounds typically represent crosscut zones of different fault systems. An example is given by the RWA cluster within the Dockweiler Wald (*cf.* Figure 10(d)), where 150 RWA mounds occurred on 1 km² only. Compared to the Oberehe site, the Dockweiler Wald RWA cluster showed manually inferred RWA Lineaments in NNE-SSW (approx. 20°), NE-SW (approx. 50–60°), WNW-ESE (approx. 110°), NW-SE (approx. 125°) and N-S (approx. 175°) direction (*cf.* Figure 10(e)). The results of the Hough transform showed similar directions: N-S (0°), NNE-SSW (39°), NE-SW (48°–63°), WNW-ESE (99°), NW-SE (126°) and NNW-SSE (165°–175°) (*cf.* Figure 10(f)).

**Figure 11:**
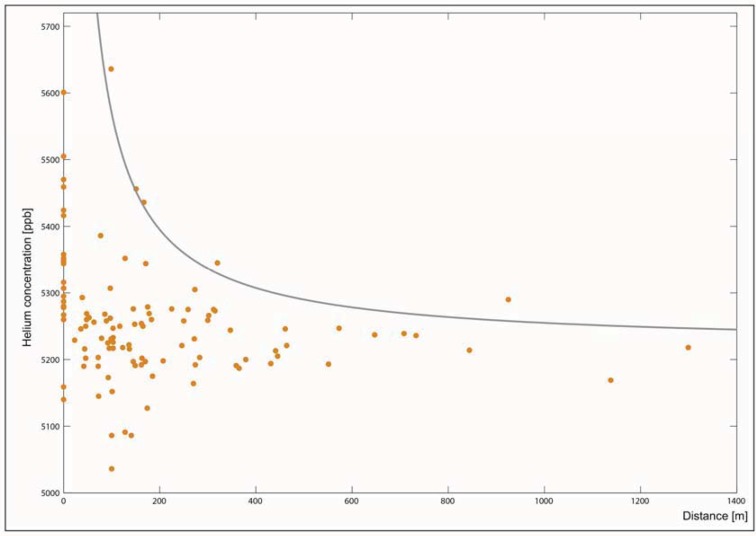
Helium concentration (ppb) *versus* distance (m) from the closest fault.

**Figure 12:**
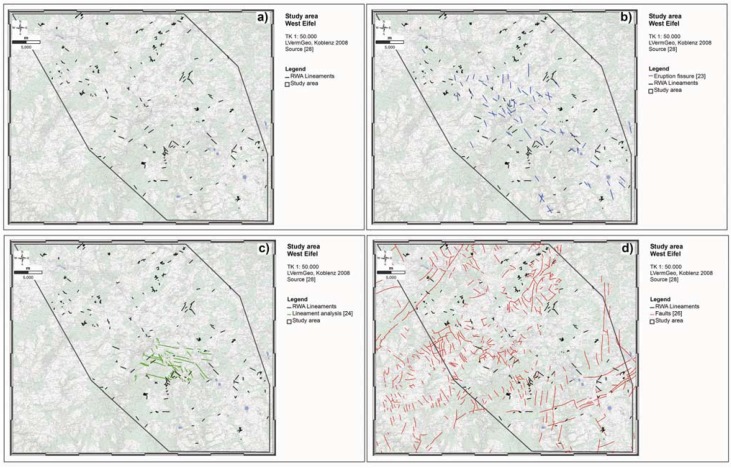
RWA Lineaments (grey lines) (**a**) combined with published tectonic features like Quaternary eruption fissures (blue lines) (**b**), lineament analyses of tectonically active faults (green lines) (**c**) and fault systems (red lines) (**d**) without any differentiation between inactive and active fault systems, for the entire study area.

For the Oberehe site, the maximum Helium concentration [ppb] (*cf.* Figure 11) for a given distance *x* from the closest fault clearly shows a decreasing trend with increasing value of *x*. High He concentrations only occur in close proximity to the faults. The comparison of both the spatial RWA mound distribution with the significantly increased geogenic gas anomalies that are controlled by macro- and micro-scale brittle deformation confirms that the RWA colony is situated in a tectonically sheared region. Their preferential linear alignment directions directly match tectonically active, gaspermeable strike-slip faults (*cf.* Figure 12(a)). Although the Oberehe reference site is surrounded by various forest stands, no further RWA mounds were found in those forest stands during the area-wide mapping. Clusters of RWA mounds typically represent crosscut zones of different fault systems. Analysis of their internal structures will similarly lead to linear alignments that depict a more complex tectonic situation, *i.e.*, voids or a transtensional/transpressional regime at that site. Another prominent example are the three large clusters of the *F. rufa* supercolony at the Bodanrück (South-West Germany), where within the clusters diffuse degassing of geogenic tracer gases occurs [65,66]. Solitary RWA mounds can be found mostly at unusual locations with spotty degassing and are links between linear alignments and cluster structures. For myrmecologists, the causes and stringency of such a linkage are paramount, since linear patterns have been mostly associated with edge effects of forest stands and/or roads [48,49]. But [40] already stated in 1929 that there are no preferred locations of RWA mounds within a forest stand. This is strengthened by statistical analyses, which show that spatial distribution of RWA map tectonically active, gaspermeable strike-slip faults [31].

These results suggest that RWA mounds can be used as biological indicators of active, gaspermeable faults [28,31,36]. This is especially useful when information about the active tectonic regime is incomplete or the resolution by technical means is insufficient [31,64,65]. Published maps with faults zones do not distinguish between inactive and active fault systems. Here, mapped RWA mounds can be used to delimit buried small-scale, tectonically active fault structures and their strike direction. These faults may be also potential earthquake areas and are simultaneously information channels deeply reaching into the crust [74,75].

## 4. Conclusions

An area wide GeoBioScience-approach in a 1,140 km² study area in the West Eifel (West Germany) demonstrated the correlation of soil gas anomalies and spatial distribution of red wood ant (RWA) mounds along tectonically active, gas-permeable faults. More specifically, the azimuthal directions in which the RWA mound positions are aligned correspond to those of the main fault directions. The presence and spatial distribution of the RWA mounds at the reference site Oberehe and in the entire study area are valuable biological indicators for a tectonically sheared area. Linear alignments of RWA mounds directly depict the fault zone. RWA clusters are typically considered to represent crosscut zones of different fault systems, reflecting the large-scale fault regime in the smallest space. These RWA mound aggregations can be used to delimit buried small-scale tectonic structures of tectonically active systems especially in those cases, where information about the active tectonic regime is incomplete or the resolution by technical means of other geological and/or geophysical methods is insufficient. Furthermore, soil gas anomalies indicate particularly favorable settlement conditions for RWA. Their mounds can only be mapped where geogenic gas anomalies occur. These results also contribute to complement to further understand settlement processes, mound start-up and spatial distribution of RWA. 
